# State Medical Association of Texas

**Published:** 1904-02

**Authors:** F. Paschal

**Affiliations:** President State Medical Association; San Antonio, Texas


### Society Notes.

### State Medical Association of Texas.

San Antonio, Texas, January 20, 1904.

*Dr. F. E. Daniel, Austin, Texas.*

Dear Doctor: Will you please announce in your valued journal that Dr. J. T. Moore, of Galveston, Texas, has been appointed Secretary of the Medical Association of Texas, to fill the unexpired term of Dr. H. A. West, deceased?

Dr. J. T. Moore has resigned as councilor of the Ninth District; Dr. H. A. Decherd, of Galveston, has been appointed in his place. Dr. W. B. Russ, of San Antonio, Texas, has been appointed Chairman of the Board of Councilors; Dr. H. B. Decherd, Secretary of of the Board of Councilors.

Dr. Wm. Gammon, Galveston, Texas, has been appointed Chairman of the Section of Pathology, vice Thos. P. Lloyd removed from the State.

Yours truly,

F. Paschal,

President State Medical Association.

